# Federal Policy Changes and Career Stability Among NIH K-Award Recipients

**DOI:** 10.1001/jamanetworkopen.2026.8554

**Published:** 2026-04-22

**Authors:** Daniel Shalev, Molly Nowels, Rose Carlson, Maureen Ekwebelem, Catherine A. Riffin, M. Carrington Reid

**Affiliations:** 1 Division of Geriatrics and Palliative Medicine, Weill Cornell Medicine, New York, New York

## Abstract

**Importance:**

Federal policy changes, including restrictions on research topics and proposed National Institutes of Health (NIH) budget reductions, may affect perceptions of career stability among early-career biomedical researchers.

**Objective:**

To evaluate how NIH K awardees perceive the effects of federal policy on research career stability and intentions to pursue independent NIH funding.

**Design, Setting, and Participants:**

In this cross-sectional survey study, an anonymous online national survey was administered from April 15 to May 27, 2025, via REDCap using contact information from the NIH RePORTER database. All principal investigators were holding individual mentored K awards initially funded between 2019 and 2025. Data were analyzed from June 11, 2025, to February 11, 2026.

**Exposure:**

Self-reported perceptions of recent federal policy changes affecting research funding and institutional support.

**Main Outcomes and Measures:**

The main outcome was perceived likelihood of remaining in science and significant funding disruptions measured via an online national survey. Secondary outcomes included perceived institutional support and the likelihood of applying for R01-equivalent funding.

**Results:**

Of 6118 K award recipients, 1904 respondents representing all NIH institutes completed the survey (adjusted response rate, 34%); 1230 (65%) identified as women and 366 (19%) identified as underrepresented in biomedical research by race, ethnicity, or disability. A total of 1819 (96%) reported that federal policy changes negatively affected the stability of their research careers. Compared with 1 year prior, 988 (52%) believed they were somewhat less likely and 341 (18%) much less likely to continue conducting research. Significant funding disruptions were reported by 343 (18%). Postdoctoral researchers were more likely than associate professors to report being much less likely to stay in science (adjusted odds ratio [aOR], 2.57; 95% CI, 1.35-4.88; *P* < .001). Respondents identifying as disabled (aOR, 2.21; 95% CI, 1.38-3.54; *P* < .001), American Indian or Alaska Native (aOR, 5.32; 95% CI, 1.01-28; *P* = .048), Black (aOR, 2.48; 95% CI, 1.52-4.05; *P* < .001), or Hispanic (aOR, 2.67; 95% CI, 1.78-3.98; *P* < .001) were more likely to report significant funding disruptions. A total of 506 respondents (27%) reported high institutional support; 523 of 1338 (39%) who had not yet applied for an R01 reported decreased likelihood of doing so.

**Conclusions and Relevance:**

In this cross-sectional survey study of NIH K-award recipients, nearly all respondents reported a decrease in career stability, and many reported reduced intent to pursue independent NIH funding amid federal policy shifts. These findings indicate widespread perceived instability among early-career NIH-funded investigators.

## Key Points

**Question:**

How are federal policy changes associated with early-career National Institutes of Health (NIH)–funded investigators’ perceptions of career stability?

**Findings:**

In this cross-sectional study of 1904 NIH K-award recipients, 18% reported being much less likely to continue conducting research than 1 year prior, and 18% reported funding disruptions. Respondents identifying as members of underrepresented racial or ethnic groups or as disabled were more likely to report career instability.

**Meaning:**

This cross-sectional study suggests strain in the biomedical research workforce as a result of federal policy changes.

## Teaser

This cross-sectional study uses survey data to evaluate how National Institutes of Health (NIH) K awardees perceive the effects of federal policy on research career stability and intentions to pursue independent NIH funding.

## Introduction

Over the past year, the US biomedical research ecosystem has been subject to significant volatility due to shifts in federal policy.^1^ Early-career investigators (ECIs; investigators who have not yet obtained an independent National Institutes of Health [NIH] award and are within 10 years of their terminal research degree or clinical training^2^) are critical to the continuation of the US research ecosystem yet occupy a particularly precarious position within this environment. They are expected to establish independent laboratories or clinical research programs while simultaneously navigating shrinking paylines, rising living costs, and limited institutional support. ECIs function as a bellwether population: compared with senior faculty, they are more vulnerable to structural policy shifts and often operate with fewer institutional protections and more precarious funding.^3,4,5,6^ Evaluating their career trajectories is therefore essential for monitoring the health of the biomedical research ecosystem and predicting its long-term stability. Their career decisions, whether to persist in NIH-funded science, disengage, or transition to other types of work, can serve as early signals of strain within the system. Attrition among this group signals not only individual loss but also potential downstream erosion of the nation’s capacity for innovation, discovery, and public health advancement.

The NIH K-series career development awards represent one of the federal government’s most substantial investments in ECIs. These awards provide protected time, structured mentorship, and salary support to facilitate the transition from mentored to independent investigator.^7^ Recipients are competitively selected for their potential to make substantial contributions to biomedical research and collectively represent a major federal investment in the next generation of researchers. Given their role in sustaining the biomedical research pipeline, the perspectives of NIH K awardees on career stability are essential to assess. If this cohort, selected for its potential to make meaningful contributions to biomedical research, leaves academic research or reconsiders applying for NIH funding, it may portend larger disruptions to the biomedical research enterprise.

Prior studies have documented persistent challenges for ECIs, including limited bridge funding, inadequate mentorship, and institutional cultures that undervalue early-stage scholars.^5,6^ Yet few, if any, studies have examined the influence of federal science policy on these challenges. Recent shifts in federal policy priorities, including changes affecting diversity initiatives and certain areas of research, represent system-level stressors distinct from traditional funding-cycle variability. These stressors may also have disproportionate effects on scientists from underrepresented racial and ethnic groups, individuals with disabilities, and those working in fields more likely to intersect with topics such as reproductive health or health equity. Existing federal workforce reports tend to focus on numerical outputs (grants awarded, publications produced) without interrogating the structural climate in which these careers unfold. Understanding this climate is essential to predicting not only who will remain in federally funded research but also the robustness and breadth of the US biomedical research ecosystem going forward.

To address this gap, we conducted a national survey of NIH K01, K08, K22, K23, K25, K38, K43, K76, and K99/R00 award recipients. Our objective was to assess how federal policy is shaping this cohort’s perceptions of career stability, decisions to apply for independent (ie, R01 or equivalent level) NIH funding, and intentions to remain in academic research.

## Methods

### Study Design and Participants

We conducted a national, cross-sectional, anonymous survey of NIH K awardees between April and June 2025. Results are reported in accordance with the Strengthening the Reporting of Observational Studies in Epidemiology (STROBE) reporting guideline for cross-sectional studies.^8^ The study was approved by the Weill Cornell Medicine institutional review board, which granted a waiver for informed consent because of the minimal risk of harm to patients and the absence of personally identifiable information. Race was assessed via self-report to examine whether members of racial and ethnic groups underrepresented in biomedical research were disproportionately affected by federal policy changes. Categories included American Indian or Alaska Native, Asian, Black, Native Hawaiian or Other Pacific Islander, White, multiracial, and prefer to self-describe (a free-text option for respondents who did not identify with the listed categories).

Eligible participants were principal investigators holding individual mentored or transition K awards (K01, K08, K22, K23, K25, K38, K43, K76, and K99/R00) initially funded between 2019 and 2025. Using publicly available contact information from the NIH RePORTER database, we identified 6118 eligible awardees and distributed electronic invitations through REDCap.^9^ The initial email contained a study information sheet and survey link; reminders were sent to nonrespondents on April 29 and May 13, 2025.

### Survey Instrument

The study team developed a 34-item survey (eAppendix in Supplement 1) to assess ECIs’ perceptions of how the federal policy landscape may be affecting their career trajectories, funding intentions, and views on institutional support. It included both structured and open-ended items across 6 domains: (1) demographics and training background; (2) perceived impact of the federal policy changes on research stability; (3) funding disruptions and concerns; (4) policy changes affecting research environment; (5) career plans and likelihood of applying for R01-equivalent funding; and (6) open-ended feedback on needed supports and anticipated career changes. Response options included categorical, Likert-scale, and open-text fields. To preserve anonymity while allowing optional follow-up, participants could provide an email address through a separate, unlinked REDCap form.

### Primary Variables and Covariates

We initially prespecified the perceived impact of the existing policy landscape on the stability of respondents’ research careers as the primary dependent variable. This variable was too homogeneous for regression modeling. We therefore focused subsequent analyses on 2 related indicators of career instability: (1) reporting being much less likely to continue to conduct research than 1 year prior; and (2) reporting significant funding disruptions attributed to policy changes. These 2 binary variables served as dependent variables in multivariable logistic regressions assessing associations with demographic, disciplinary, and institutional characteristics.

Secondary analyses examined (1) decreased likelihood of applying for R01-equivalent funding and (2) perceived low institutional support (“little or none” vs “partial or high”). Prespecified covariates were chosen based on the social ecological framework, which conceptualizes career outcomes as emerging from individual, disciplinary, and institutional factors within a broader policy environment.^10^ Demographic variables included age (<35, 35-39, 40-44, and ≥45 years), gender (man, woman, nonbinary, or prefer not to say), race and ethnicity (respondents selecting >1 racial category were categorized as multiracial for analytic purposes), disability status, and political views (from very liberal to very conservative). Professional characteristics included academic rank (postdoctoral researcher or fellow, instructor, assistant professor, associate professor, research scientist, and other), K-award mechanism, primary discipline (physician, basic-science PhD, public-health PhD, social or behavioral-science PhD, clinical psychologist, multidisciplinary, and other), and award year. Institutional variables included academic medical center or R1 status and public vs private status.

### Statistical Analysis

Descriptive statistics were computed using Stata SE, version 18 (StataCorp LLC). Regressions were conducted using R, version 4.5.1 (R Foundation for Statistical Computing). Descriptive statistics summarized sample characteristics with counts and percentages for categorical variables and median (IQR) values for ordinal variables. Two multivariable logistic regression models estimated adjusted odds ratios (aORs) and 95% CIs for the 2 dependent variables described. All covariates listed were entered simultaneously. Reference categories were 45 years of age or older (age), men (gender), White (race), non-Hispanic (ethnicity), nondisabled (disability status), associate professor (rank), private institution (institutional affiliation), K01 (mechanism), and physician (discipline). Only complete cases were included in logistic regression analyses. All covariates were selected a priori based on a conceptual framework linking individual, disciplinary, and institutional factors to career stability and were retained in multivariable models regardless of statistical significance to reduce omitted-variable bias and support consistent adjustment across outcomes. All statistical tests were 2 sided with significance set at α = .05. Results are reported as aORs (95% CIs). Qualitative results and analysis were not included in this manuscript. Data were analyzed from June 11, 2025, to February 11, 2026.

## Results

### Sample Characteristics

Of the 6118 eligible NIH K awardees contacted, 1904 completed the survey (adjusted response rate, 34% after excluding 486 undeliverable emails). Item-level missingness was low (<5%); analyses were based on available responses without imputation. Respondents represented all NIH institutes and a broad range of disciplines and career stages within ECI status (Table 1).^11,12,13,14^ Overall, respondents were similar to the overall NIH K-award population with respect to demographics and award mechanism, although women were modestly overrepresented.

**Table 1.** Demographic and Professional Characteristics of Survey Respondents Compared to NIH Population Data^a^

Variable and category	No. (%)	NIH reference
Age, y (n = 1894)		
≤34	279 (15)	Mean age (SD) of applicants: K01, 37.7 (5.3) y; K08, 36.8 (4.0) y; K23 38.3 (5.4)^b^
35-39	780 (41)	
40-44	624 (33)	
≥45	211 (11)	
Gender (n = 1895)		
Men	630 (33)	NA
Women	1230 (65)	55% in 2020, 58% in 2025^c^
Other or prefer not to say	35 (2)	NA
Race (n = 1887)		
Asian	333 (18)	28%^d^
Black or African American	113 (6)	5%
White	1346 (71)	50%
Prefer not to say	80 (4)	NA
Other^e^	80 (4)	NA
Ethnicity (n = 1887)		
Hispanic or Latino/a/x	164 (9)	8%^f^
Non-Hispanic or Latino/a/x	1669 (88)	92%
Prefer not to say	54 (3)	NA
Disability status (n = 1880)		
Yes	102 (5)	2%^f^
No	1723 (92)	98%
Prefer not to say	55 (3)	NA
K-award year (n = 1816)		
2019 or earlier	105 (6)	814^g^
2020	231 (13)	895
2021	263 (15)	1181
2022	325 (18)	1267
2023	402 (22)	1170
2024	423 (23)	1141
2025	61 (3)	669
K-award mechanism (n = 1888)		
K01	477 (25)	21%^g^
K08	325 (17)	17%
K23	553 (29)	25%
K99/R00	438 (23)	31%
K22/K25/K38/K43/K76	95 (5)	6%
Discipline (n = 1892)		
PhD (basic science)	695 (37)	NA
PhD (public health or population science)	258 (14)	
PhD (social or behavioral science)	237 (13)	
PhD (other)	118 (6)	
Physician	677 (36)	
Clinical psychologist	166 (9)	
Other	140 (7)	
Clinical practice (n = 1892)		
Yes	859 (45)	NA
No	1033 (55)	
Position (n = 1898)		
Postdoctoral researcher or fellow	223 (12)	NA
Assistant professor	1217 (64)	
Associate professor	262 (14)	
Instructor	106 (6)	
Other	90 (5)	
Institutional affiliation (n = 1895)		
Academic medical center	1319 (70)	NA
R1 institution	866 (46)	
R2 institution	19 (1)	
Private and/or nonprofit research institution	132 (7)	
Other	31 (2)	
Political views (n = 1883)		
Very liberal or somewhat liberal	1282 (68)	NA
Moderate	391 (21)	
Very conservative or somewhat conservative	59 (3)	
Other or prefer not to say	140 (7)	

Abbreviations: NA, not available; NIH, National Institutes of Health.

^a^ Percentages reflect the proportion of respondents within each demographic or training category. National comparison data were obtained from publicly available NIH Data Book and RePORTER reports; some variables (eg, discipline, political views, and institutional type) are not publicly available at the individual awardee level.

^b^ Data specific to NIH Career Development Awards (K01, K07, K08, K22, K23, K25, K99, KL1, and KL2).^11^

^c^ Data specific to NIH Career Development Awards.^12^

^d^ Data specific to NIH Career Development Awards.^13^

^e^ American Indian or Alaska Native; Native Hawaiian or Other Pacific Islander; or prefer to self-describe.

^f^ Data regarding all NIH research project awards, including career development awards.^12^

^g^ Number of new awards.^14^

Among respondents, 1230 (65%) identified as women, 630 (33% ) as men, and 35 (2%) as nonbinary, self-described, or preferred not to state gender. A total of 1346 respondents (71%) identified as White and 1669 (88%) as non-Hispanic. There were 366 participants (19%) who identified as underrepresented in biomedical research by race (American Indian or Alaska Native, Black or African American, and Native Hawaiian and Other Pacific Islander), ethnicity (Hispanic or Latino), or having a disability. Forty-four US states, as well as Puerto Rico and Washington, DC, were represented (Figure), with California (224 [12%]) and New York (174 [10%]) being the most frequent.

**Figure.**
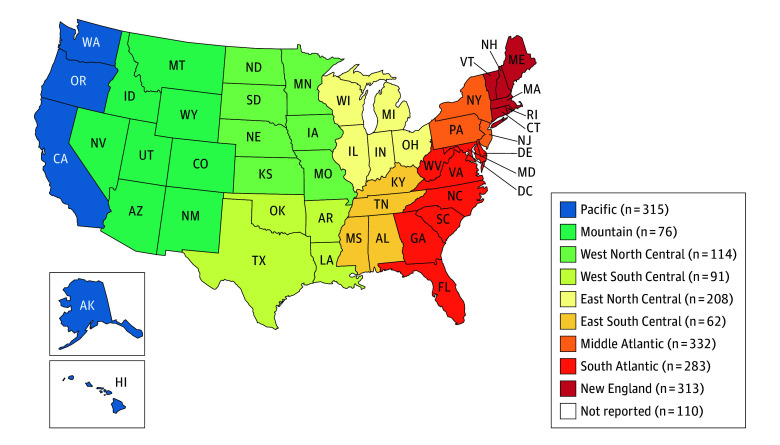
Map Showing the Geographic Distribution of Respondents

Among respondents, 1217 (64%) were assistant professors, 223 (12%) were postdoctoral researchers or fellows, and 262 (14%) were associate professors. Most (1319 [70%]) were based at academic medical centers, and nearly half (866 [46%]) reported affiliation with R1 institutions. Approximately half (923 [48%]) of respondents were in clinical disciplines (a composite of physicians, clinical psychologists, dentists, nurses, social workers, and physical therapists).

The most frequent award mechanisms were K23 (553 [29%]), K01 (477 [25%]), and K99/R00 (438 [23%]). The most represented funding institutes were the National Heart, Lung, and Blood Institute (232 [12%]), the National Institute of Diabetes and Digestive and Kidney Diseases (186 [10%]), and the National Institute on Aging (175 [9%]). See Table 1 for full details.

### Perceptions of Career Stability

Perceptions of instability were nearly universal. Overall, 96% of respondents reported that existing policy had negatively affected the stability of their research career (very negatively, 63%; somewhat negatively, 33%). Because of this homogeneity, the variable was excluded from the regression analyses.

Compared with 1 year earlier, 988 (52%) reported feeling somewhat less likely, and 341 (18%) much less likely, to continue conducting research. Furthermore, 486 (26%) of respondents reported no change in their likelihood of continuing to conduct research. Only 86 (5%) reported a somewhat or much greater likelihood of continuing to conduct research. The most common factors influencing this perception were funding instability (1766 [95%]), political interference in science (1355 [73%]), and governmental restrictions on research topics (1166 [63%]) (eTable in Supplement 1). Bivariate associations between respondent characteristics and their reporting to be much less likely to remain in science are shown in Table 2.

**Table 2.** Characteristics of K Awardees Overall and by “Leaving Science” and “Funding Disruption” Status

Category	Much less likely to continue conducting research, No. (%)		χ^2^ Test *P* value	Experienced significant funding disruptions, No. (%)		χ^2^ Test *P* value
	No (n = 1563)	Yes (n = 341)		No (n = 1561)	Yes (n = 343)	
Age group, y						
<35	224 (14)	55 (16)	.17	213 (14)	66 (19)	.06
35-39	658 (42)	122 (36)		650 (42)	130 (38)	
40-44	506 (32)	118 (35)		517 (33)	107 (31)	
≥45	167 (11)	44 (13)		172 (11)	39 (11)	
Missing	8 (1)	2 (1)		9 (1)	1 (<1)	
Gender						
Men	512 (33)	118 (35)	.72	524 (34)	106 (31)	.34
Prefer to self-describe, prefer not to say, or nonbinary	29 (2)	6 (2)		26 (2)	9 (3)	
Women	1018 (65)	212 (63)		1003 (65)	227 (66)	
Missing	4 (<1)	5 (1)		8 (1)	1 (<1)	
Race						
American Indian or Alaska Native	4 (<1)	3 (1)	.047	3 (<1)	4 (1)	<.001
Asian	253 (16)	45 (13)		246 (16)	52 (16)	
Black	88 (6)	14 (4)		71 (5)	31 (9)	
Multiracial^a^	48 (3)	15 (4)		47 (3)	16 (5)	
White	1064 (69)	227 (68)		1082 (70)	209 (62)	
Other^b^	95 (6)	31 (9)		102 (7)	24 (7)	
Missing	11 (1)	6 (2)		10 (1)	7 (2)	
Ethnicity						
Hispanic	128 (8)	36 (11)	.19	107 (7)	57 (17)	<.001
Non-Hispanic or other	1422 (92)	301 (89)		1441 (93)	282 (83)	
Missing	13 (1)	4 (1)		13 (1)	4 (1)	
Disability status						
Not disabled or other	1468 (95)	310 (92)	.05	1472 (96)	306 (90)	<.001
Disabled	76 (5)	26 (8)		69 (4)	33 (10)	
Missing	19 (1)	5 (1)		20 (1)	4 (1)	
Professional position						
Associate professor	213 (13)	49 (14)	.06	218 (14)	44 (13)	<.001
Assistant professor	1018 (65)	199 (59%)		1020 (66)	197 (58)	
Instructor	89 (6)	17 (5)		90 (6)	16 (5)	
Research scientist or other	26 (2)	11 (3)		30 (2)	7 (2)	
Postdoctoral researcher/fellow	173 (11)	50 (15)		153 (10)	70 (21)	
Research scientist	40 (3)	13 (4)		45 (3)	8 (2)	
Missing	4 (<1)	2 (1)		5 (<1)	1 (<1)	
Academic medical center or R1 institution						
Yes	1481 (95)	327 (96)	.46	1482 (95)	326 (95)	.99
No	82 (5)	14 (4)		79 (5)	17 (5)	
Public institution						
No	1056 (68)	219 (64)	.26	1031 (66)	244 (71)	.08
Yes	507 (32)	122 (36)		530 (34)	99 (29)	
K-award type						
K01	396 (25)	81 (24)	.08	396 (25)	81 (24)	<.001
K08	262 (17)	63 (19)		274 (18)	51 (15)	
K23	440 (28)	113 (33)		484 (31)	69 (20)	
K99/R00	365 (23)	73 (21)		310 (20)	128 (37)	
Other	100 (6)	11 (3.2)		97 (6)	14 (4)	
Discipline						
Physician	389 (25)	103 (30)	<.001	425 (27)	67 (20)	<.001
Basic science PhD	418 (27)	77 (23)		369 (24)	126 (37)	
Clinical psychologist	81 (5)	27 (8)		89 (6)	19 (6)	
Multidisciplinary	306 (20)	79 (23)		323 (21)	62 (18)	
Other	23 (1)	2 (1)		19 (1)	6 (2)	
Other clinician	21 (1)	0		19 (1)	2 (1)	
Other PhD	66 (4)	2 (0.6)		57 (4)	11 (3)	
Population or public health PhD	145 (9)	34 (10)		148 (10)	31 (9)	
Social or behavioral science PhD	104 (7)	15 (4)		102 (7)	17 (5)	
Missing	10 (1)	2 (1)		10 (1)	2 (1)	

^a^ Respondents selecting more than 1 racial category.

^b^ Includes Native Hawaiian or Other Pacific Islander and those who preferred to self-describe.

In multivariable modeling (Table 3), postdoctoral investigators (aOR, 2.57; 95% CI, 1.35-4.88; *P* < .001) and individuals in non–tenure-track positions (postdoctoral researchers, research scientists, and instructors) had higher odds of reporting being much less likely to remain in science compared with associate professors. The aOR for respondents identifying as disabled was greater than 1, but the finding was not statistically significant (aOR, 1.61; 95% CI, 0.98-2.65; *P* = .06), whereas investigators with nonclinical PhD training did not demonstrate increased likelihood of reporting plans to leave research (basic science: aOR, 0.59; 95% CI, 0.35-1.00; *P* = .05; other PhD: aOR, 0.20; 95% CI, 0.03-0.51; *P* < 001; public health: aOR, 0.84; 95% CI, 0.46-1.54; *P* = .57; behavioral science: aOR, 0.53; 95% CI, 0.27-1.04; *P* = .06).

**Table 3.** Multivariable Logistic Regression Results for “Much Less Likely to Continue Conducting Research”

Category	aOR (95% CI)	*P* value
Age group, y		
<35	0.82 (0.48-1.41)	.48
35-39	0.64 (0.42-0.99)	.047
40-44	0.79 (0.52-1.19)	.26
≥45	1 [Reference]	NA
Gender		
Nonbinary or prefer not to say	0.72 (0.25-2.07)	.54
Men	1 [Reference]	NA
Women	0.86 (0.66-1.13)	.28
Race		
American Indian or Alaska Native	2.49 (0.52-11.95)	.25
Asian	0.83 (0.56-1.21)	.32
Black	0.84 (0.46-1.56)	.59
Multiracial	1.21 (0.64-2.29)	.56
White	1 [Reference]	NA
Other^a^	1.35 (0.80-2.28)	.26
Ethnicity		
Hispanic	1.24 (0.78-1.92)	.34
Non-Hispanic	1 [Reference]	NA
Disability status		
Disabled	1.61 (0.98-2.65)	.06
Not disabled	1 [Reference]	NA
Position		
Assistant professor	0.95 (0.65-1.4)	.81
Instructor	1.28 (0.66-2.48)	.47
Other	2.60 (1.12-6.02)	.03
Postdoctoral researcher or fellow	2.57 (1.35-4.88)	<.001
Research scientist	1.64 (0.74-3.65)	.22
Associate professor	1 [Reference]	NA
Academic medical center		
No	0.83 (0.44-1.59)	.58
Yes	1 [Reference]	NA
Public institution		
Yes	1.18 (0.90-1.55)	.23
No	1 [Reference]	NA
K mechanism		
K01	1 [Reference]	NA
K08	0.87 (0.54-1.42)	.58
K23	1.00 (0.63-1.60)	.99
K99/R00	0.67 (0.40-1.12)	.13
Other K	0.52 (0.26-1.07)	.08
Discipline		
Physician	1 [Reference]	NA
Basic science	0.59 (0.35-1.00)	.05
Clinical psychology	1.46 (0.87-2.48)	.15
Multidisciplinary	0.96 (0.65-1.41)	.83
Other	0.30 (0.06-1.39)	.12
Other clinician	0.00 (0.00-4.45)	.96
Other PhD	0.12 (0.03-0.51)	<.001
Public health	0.84 (0.46-1.54)	.57
Social or behavioral science	0.53 (0.27-1.04)	.06
Political views		
Very liberal	1 [Reference]	NA
Somewhat liberal	0.90 (0.66-1.22)	.49
Moderate	1.06 (0.73-1.54)	.75
Somewhat conservative	0.89 (0.39-2.04)	.79
Very conservative	0.60 (0.07-5.28)	.64
Libertarian	3.02 (0.82-11.16)	.10
Other	2.14 (0.96-4.80)	.06
Prefer not to say	0.99 (0.54-1.81)	.96

Abbreviations: aOR, adjusted odds ratio; NA, not applicable.

^a^ Includes Native Hawaiian or Other Pacific Islander and those who preferred to self-describe.

Perceptions of institutional support were mixed: 506 (27%) believed their institution was highly supportive, 854 (44%) reported partial or inconsistent support, and 429 (23%) reported little to no support. A minority (118 [6%]) were unsure about their institution’s level of support .

### Funding Disruptions

Overall, 805 respondents (42%) reported funding disruptions, including 343 (18%) reporting significant disruptions and 462 (24%) reporting minor disruptions. An additional 1050 (55%) reported no disruptions but expressed concern that disruptions might occur, and 47 (2%) reported no disruptions or concerns. Bivariate associations between respondent characteristics and reporting significant funding disruptions are shown in Table 2.

In multivariable modeling (Table 4), respondents identifying as American Indian or Alaska Native (aOR, 5.32; 95% CI, 1.01-28; *P* = .048), Black (aOR, 2.48; 95% CI, 1.52-4.05; *P* < .001), or Hispanic (aOR, 2.67; 95% CI, 1.78-3.98; *P* < .001), as well as those identifying as disabled (aOR, 2.21; 95% CI, 1.38-3.54; *P* < .001), had higher odds of reporting significant funding disruptions compared with their reference groups. Individuals reporting nonbinary or unspecified gender identity also demonstrated increased odds (aOR, 3.15; 95% CI, 1.24-8.02; *P* = .02). In contrast, K23 awardees had lower odds of reporting disruptions compared with K01 awardees (aOR, 0.61; 95% CI, 0.37-0.99; *P* = .04), whereas K99/R00 recipients had higher odds (aOR, 1.88; 95% CI, 1.22-2.9; *P* < .001).

**Table 4.** Multivariable Logistic Regression Results for Having Experienced “Significant Funding Disruptions”

Category	aOR (95% CI)	*P* value
Age group, y		
<35	0.84 (0.49-1.45)	.53
35-39	0.84 (0.53-1.32)	.45
40-44	0.99 (0.64-1.54)	.97
≥45	1 [Reference]	NA
Gender		
Men	1 [Reference]	NA
Nonbinary or prefer not to say	3.15 (1.24-8.02)	.02
Women	1.23 (0.92-1.63)	.16
Race		
American Indian or Alaska Native	5.32 (1.01-28.00	.048
Asian	1.17 (0.81-1.68)	.41
Black	2.48 (1.52-4.05)	<.001
Multiracial	1.58 (0.84-2.97)	.16
White	1 [Reference]	NA
Other^a^	0.72 (0.41-1.26)	.25
Ethnicity		
Hispanic	2.67 (1.78-3.98)	<.001
Non-Hispanic	1 [Reference]	NA
Disability status		
Disabled	2.21 (1.38-3.54)	<.001
Not disabled	1 [Reference]	NA
Position		
Assistant professor	0.80 (0.53-1.21)	.29
Instructor	0.61 (0.30-1.25)	.18
Other	0.78 (0.30-2.06)	.62
Postdoctoral researcher	1.01 (0.55-1.83)	.98
Research scientist	0.63 (0.26-1.54)	.31
Associate professor	1 [Reference]	NA
Academic medical center		
No	0.91 (0.50-1.66)	.76
Yes	1 [Reference]	NA
Public institution		
Yes	1.02 (0.76-1.35)	.91
No	1 [Reference]	NA
K mechanism		
K01	1 [Reference]	NA
K08	0.89 (0.54-1.46)	.64
K23	0.61 (0.37-0.99)	.04
K99/R00	1.88 (1.22-2.90)	<.001
Other K	0.69 (0.36-1.32)	.26
Discipline		
Physician	1 [Reference]	NA
Basic science	1.00 (0.59-1.68)	.99
Clinical psychology	1.30 (0.71-2.39)	.39
Multidisciplinary	0.88 (0.57-1.37)	.58
Other	1.43 (0.47-4.39)	.53
Other clinician	0.44 (0.09-2.03)	.29
Other PhD	0.77 (0.35-1.71)	.52
Public health	0.64 (0.34-1.21)	.17
Behavioral science	0.68 (0.34-1.34)	.26
Political views		
Very liberal	1 [Reference]	NA
Somewhat liberal	0.97 (0.71-1.33)	.87
Moderate	1.09 (0.75-1.59)	.64
Somewhat conservative	0.91 (0.42-1.97)	.80
Very conservative	0.65 (0.07-5.81)	.70
Libertarian	0.65 (0.12-3.66)	.63
Other	1.76 (0.77-4.02)	.18
Prefer not to say	0.77 (0.42-1.44)	.42

Abbreviations: aOR, adjusted odds ratio; NA, not applicable.

^a^ Includes Native Hawaiian or Other Pacific Islander and those who preferred to self-describe.

### Plans to Submit an R01

Of the respondents, 566 (30%) had already submitted an R01, 1117 (59%) planned to submit one within the next 3 years, and 214 (11%) were unsure or did not plan to apply for an R01-equivalent grant. Of the 1338 respondents who had not yet applied for an R01, 523 (39%) reported being less likely to apply than they were 1 year prior, whereas only 103 (8%) reported a greater likelihood. In a separate survey item assessing plans for R01-equivalent submission, 214 respondents (11%) indicated that they either were uncertain about or did not plan to apply. Among these respondents, the most frequently selected reasons were concerns about funding career stability (164 [77%]), perceptions of government policies affecting scientific research (133 [62%]), and intentions to transition out of academic research ( 80 [37%]).

## Discussion

In this national cross-sectional survey of NIH K awardees, the overwhelming majority perceived recent policy changes as negatively affecting their career stability, with many indicating a decreased likelihood of continuing to conduct research. Funding instability, political interference, and restrictions on research topics were frequently cited causes. Although many respondents planned to apply for R01-equivalent grants, a substantial proportion reported less certainty about doing so compared with 1 year ago.

The near-universal perception of career instability among K awardees underscores substantial concern within the early-career biomedical research workforce. Respondents frequently cited concerns about restrictions on research topics and funding limitations.

Regression analyses further highlighted disproportionate effects across groups that have historically been underrepresented in science. Respondents identifying as members of underrepresented racial or ethnic groups, as disabled, or as nonbinary or unspecified gender were more likely to report significant funding disruptions and reduced confidence in career stability. These findings align with prior literature on inequities in NIH funding success and underscore the risk that policy volatility may exacerbate existing representation gaps.^15^ Postdoctoral researchers and individuals in non–tenure-track positions reported greater vulnerability, suggesting that structural precarity at early career stages may increase risk of leaving research, whereas several PhD-only disciplines did not demonstrate increased risk. Of note, political ideology was not independently associated with respondents reporting being much less likely to remain in science or experiencing significant funding disruptions.

These findings build on prior data documenting burnout, attrition, and limited institutional support among ECIs, but within the specific context of an unusually volatile environment for biomedical research.^5,6,16^ Our findings suggest that, in addition to traditional structural barriers (eg, limited access to mentorship, low funding rates, and differential institutional support), the broader scientific policy climate has become a significant and powerful determinant of perceived career viability and funding stability for ECIs.^6,16^ The erosion of confidence among K awardees, a group that has already secured competitive federal support, signals broader fragility across the biomedical research pipeline. Several mechanisms may underlie these perceptions, including institutional financial instability tied to shifting federal priorities, uncertainty regarding fundable research topics, and erosion of institutional supports historically benefiting underrepresented investigators.^17,18^

These findings highlight the need for coordinated action at both federal and institutional levels to sustain the early-career biomedical research workforce. Federal agencies can strengthen confidence by providing transparent communication about funding trajectories, reaffirming the independence of peer review processes underlying funding decisions and safeguarding research on topics that have become contested in policy. Institutions, meanwhile, can mitigate uncertainty by creating bridge-funding mechanisms, offering clear guidance on permissible research under evolving regulations, and visibly supporting scholars working in contested topics. The disproportionate burden reported by underrepresented investigators is particularly concerning. The existing policy environment risks exacerbating the persistent funding disparities experienced by investigators who are underrepresented in the sciences.^15,19^ Without targeted interventions, decades of progress toward workforce diversity and innovation may be undermined.

Future research should examine whether and how these perceptions of instability and policy challenges influence career trajectories. Longitudinal studies following ECIs over time could clarify whether perceived threats to the research climate translate into reduced research engagement or attrition from the biomedical research workforce. It will also be important to assess whether similar patterns are observed among midcareer and senior investigators, who may experience and respond to these pressures differently. Comparative and repeated assessments across career stages could help identify vulnerable inflection points in the research pipeline and inform targeted policy and institutional responses.

### Strengths and Limitations

Strengths of this study include its large, diverse, and nationally representative sample of federally funded ECIs, as well as one of the most comprehensive surveys of the NIH K-award population to date. Respondents represented all major NIH institutes and a broad range of disciplines, funding mechanisms, and geographic regions, allowing for the assessment of both individual- and institutional-level factors. The sampling frame, derived directly from publicly available NIH RePORTER data, enhances transparency and reproducibility and minimizes selection bias related to recruitment networks or professional societies.

The study design also captures a uniquely critical moment in the US scientific enterprise: data were collected during an interval of significant policy flux and scrutiny of academic research. By quantifying K awardees’ perceptions during this acute period, our findings provide an early empirical signal of how policy volatility may influence retention, motivation, and perceived viability among the next generation of independent investigators.

Limitations include the cross-sectional design, which precludes causal inference; reliance on self-report, which may be influenced by social desirability bias; and a modest response rate, raising the possibility of nonresponse bias. It is possible that investigators who were more affected by recent policy changes were more likely to respond, thereby overestimating the prevalence of perceived instability. Conversely, investigators experiencing acute disruption may have lacked time to respond, potentially biasing results in the opposite direction. We did not formally examine whether individuals underrepresented in science were disproportionately geographically clustered. Regional variation in institutional funding environments or state-level policy contexts may partially influence perceptions of instability and funding disruption. Future work incorporating regional-level analyses could further clarify these associations. The homogeneity of the central policy climate variable limited its analytic utility, although this itself conveys the consensus of perceived instability. Unmeasured institutional characteristics may still confound observed associations.

## Conclusions

In this cross-sectional survey study of self-reported career stability of NIH K awardees, respondents overwhelmingly perceived the 2025 federal policy climate as destabilizing to their research careers. The uniformity of concern among this select group of federally funded scientists suggests a broader challenge to the stability and diversity of the US research enterprise. Ensuring stability of early-career research support across changing federal policy environments will be essential to preserving the nation’s capacity for biomedical innovation and discovery.

## Notes

Supplement 1. **eAppendix.** Survey Instrument **eTable.** Tabulation of Factors Influencing Perceived Likelihood of Continuing to Conduct Science

Supplement 2.
Data Sharing Statement
