# Supplementary material for: Federal Policy Changes and Career Stability Among NIH K-Award Recipients
Source: JAMA Netw Open. 2026 Apr 22;9(4):e268554. doi: 10.1001/jamanetworkopen.2026.8554 (PMC13103800; doi:10.1001/jamanetworkopen.2026.8554)
Supplement: Supplement 1. — eAppendix. Survey Instrument eTable. Tabulation of Factors Influencing Perceived Likelihood of Continuing to Conduct Science [file jamanetwopen-e268554-s001.pdf]

# Supplemental Online Content

Shalev D, Nowels M, Carlson R, Ekwebelem M, Riffin CA, Reid MC. Federal policy changes and career stability among NIH K-award recipients. *JAMA Netw Open*. 2026;9(4):e268554. doi:10.1001/jamanetworkopen.2026.8554

**eAppendix.** Survey Instrument
**eTable.** Tabulation of factors influencing perceived likelihood of continuing to conduct science

This supplemental material has been provided by the authors to give readers additional information about their work.

© 2026 Shalev D. *JAMA Network Open.*

**eAppendix: Survey Instrument**

© 2026 Shalev D. *JAMA Network Open.*

| | |
|---|---|
| What is your age? | ◯ Under 30<br>◯ 30-34<br>◯ 35-39<br>◯ 40-44<br>◯ 45-49<br>◯ 50+ |
| What is your gender? | ◯ Man<br>◯ Woman<br>◯ Non-binary/gender nonconforming<br>◯ Prefer to self-describe<br>◯ Prefer not to say |
| Prefer to self-describe (please specify): | __________________________________ |
| What is your ethnicity? (Select all that apply) | ◯ Hispanic or Latino/a/x<br>◯ Not Hispanic or Latino/a/x<br>◯ Prefer not to say |
| What is your race? (Select all that apply) | ☐ American Indian or Alaska Native<br>☐ Asian<br>☐ Black or African American<br>☐ Native Hawaiian or Other Pacific Islander<br>☐ White<br>☐ Prefer to self-describe<br>☐ Prefer not to say |
| Prefer to self-describe (please specify): | __________________________________ |
| Do you consider yourself to have a disability as defined by the NIH? (The NIH defines disability as a physical or mental impairment substantially limiting one or more major life activities.) | ◯ Yes<br>◯ No<br>◯ Prefer not to say |
| What is your current position/title? | ◯ Postdoctoral researcher/fellow<br>◯ Instructor<br>◯ Assistant Professor<br>◯ Associate Professor<br>◯ Research Scientist<br>◯ Other (please specify) |
| Other (please specify): | __________________________________ |
| What type of institution are you affiliated with? (Select all that apply) | ☐ Academic medical center<br>☐ Government research institution (e.g., NIH, CDC<br>☐ Private and/or non-profit non-university research institution<br>☐ Industry<br>☐ R1 research institution<br>☐ R2 research institution<br>☐ Other (please specify) |
| Is your institution private or public? (Select all that apply) | ☐ Private<br>☐ Public |
| Other (please specify): | __________________________________ |

Where is your primary institution located?

- ◯ Alabama
- ◯ Alaska
- ◯ Arizona
- ◯ Arkansas
- ◯ California
- ◯ Colorado
- ◯ Connecticut
- ◯ Delaware
- ◯ District of Columbia (Washington, D.C.)
- ◯ Florida
- ◯ Georgia
- ◯ Hawaii
- ◯ Idaho
- ◯ Illinois
- ◯ Indiana
- ◯ Iowa
- ◯ Kansas
- ◯ Kentucky
- ◯ Louisiana
- ◯ Maine
- ◯ Maryland
- ◯ Massachusetts
- ◯ Michigan
- ◯ Minnesota
- ◯ Mississippi
- ◯ Missouri
- ◯ Montana
- ◯ Nebraska
- ◯ Nevada
- ◯ New Hampshire
- ◯ New Jersey
- ◯ New Mexico
- ◯ New York
- ◯ North Carolina
- ◯ North Dakota
- ◯ Ohio
- ◯ Oklahoma
- ◯ Oregon
- ◯ Pennsylvania
- ◯ Puerto Rico
- ◯ Rhode Island
- ◯ South Carolina
- ◯ South Dakota
- ◯ Tennessee
- ◯ Texas
- ◯ Utah
- ◯ Vermont
- ◯ Virginia
- ◯ Washington
- ◯ West Virginia
- ◯ Wisconsin
- ◯ Wyoming
- ◯ American Samoa
- ◯ Guam
- ◯ Northern Mariana Islands
- ◯ U.S. Virgin Islands
- ◯ Prefer not to say

© 2026 Shalev D et al. JAMA Network Open.

| | |
|---|---|
| What is your K-award mechanism? | ◯ K01<br>◯ K02<br>◯ K08<br>◯ K22<br>◯ K23<br>◯ K25<br>◯ K38<br>◯ K43<br>◯ K76<br>◯ K99/R00 |
| What NIH institute, office, or program funds your K-award? | ◯ AHRQ - Agency for Healthcare Research and Qualit<br>◯ CDC - Centers for Disease Control and Prevention<br>◯ FIC - Fogarty International Center<br>◯ NCATS - National Center for Advancing Translational Sciences<br>◯ NCCIH - National Center for Complementary and Integrative Health<br>◯ NCIPC - National Center for Injury Prevention and Control<br>◯ NCI - National Cancer Institute<br>◯ NEI - National Eye Institute<br>◯ NHGRI - National Human Genome Research Institut<br>◯ NHLBI - National Heart, Lung, and Blood Institute<br>◯ NIA - National Institute on Aging<br>◯ NIAAA - National Institute on Alcohol Abuse and Alcoholism<br>◯ NIAID - National Institute of Allergy and Infectious Diseases<br>◯ NIAMS - National Institute of Arthritis and Musculoskeletal and Skin Diseases<br>◯ NIBIB - National Institute of Biomedical Imaging and Bioengineering<br>◯ NICHD - Eunice Kennedy Shriver National Institute of Child Health and Human Development<br>◯ NIDCD - National Institute on Deafness and Other Communication Disorders<br>◯ NIDCR - National Institute of Dental and Craniofacial Research<br>◯ NIDDK - National Institute of Diabetes and Digestive and Kidney Diseases<br>◯ NIDA - National Institute on Drug Abuse<br>◯ NIEHS - National Institute of Environmental Health Sciences<br>◯ NIGMS - National Institute of General Medical Sciences<br>◯ NIMH - National Institute of Mental Health<br>◯ NIMHD - National Institute on Minority Health and Health Disparities<br>◯ NINDS - National Institute of Neurological Disorders and Stroke<br>◯ NINR - National Institute of Nursing Research<br>◯ NIOSH - National Institute for Occupational Safety and Health<br>◯ NLM - National Library of Medicine<br>◯ ODSS - Office of Data Science Strategy |

© 2026 Shalev D et al. JAMA Network Open.

| | |
|---|---|
| What year was your K award funded? | ◯ Before 2019<br>◯ 2019<br>◯ 2020<br>◯ 2021<br>◯ 2022<br>◯ 2023<br>◯ 2024<br>◯ 2025 |
| What is your primary discipline (Select all that apply)? | ☐ PhD - Basic Science<br>☐ PhD - Public Health/Population Science<br>☐ PhD - Social Behavioral Science<br>☐ PhD - Other (please specify)<br>☐ Physician<br>☐ Clinical Psychologist<br>☐ Nurse<br>☐ Physician Assistant<br>☐ Physical Therapist<br>☐ Occupational Therapist<br>☐ Social Worker<br>☐ Chaplain<br>☐ Dentist<br>☐ Other (please specify) |
| Other PhD (please specify): | __________________________________ |
| Other discipline (please specify): | __________________________________ |
| Do you currently practice clinically? | ◯ Yes<br>◯ No |

© 2026 Shalev D et al. JAMA Network Open.

| | |
|---|---|
| If you are a clinician, what is your specialty? (For physicians, please select your ACGME-recognized specialty) | ☐ Allergy and Immunology<br>☐ Anesthesiology<br>☐ Colon and Rectal Surgery<br>☐ Dermatology<br>☐ Diagnostic Radiology<br>☐ Emergency Medicine<br>☐ Family Medicine<br>☐ Internal Medicine<br>☐ Medical Genetics and Genomics<br>☐ Neurology<br>☐ Nuclear Medicine<br>☐ Obstetrics and Gynecology<br>☐ Ophthalmology<br>☐ Orthopedic Surgery<br>☐ Otolaryngology<br>☐ Pathology<br>☐ Pediatrics<br>☐ Physical Medicine and Rehabilitation<br>☐ Plastic Surgery<br>☐ Preventive Medicine<br>☐ Psychiatry<br>☐ Radiation Oncology<br>☐ Surgery<br>☐ Thoracic Surgery<br>☐ Urology<br>☐ Vascular Surgery<br>☐ Other (please specify) |
| Other (please specify): | __________________________________ |

**Section 2: Impact of Political Climate on Career Prospects**

| | |
|---|---|
| How has the current political climate affected your perception of long-term stability in your research career? | ◯ Very negatively<br>◯ Somewhat negatively<br>◯ Neutral<br>◯ Somewhat positively<br>◯ Very positively |
| Do you feel your institution is providing sufficient support to K-awardees during this time? | ◯ Yes, my institution is highly supportive<br>◯ Somewhat, my institution provides some support bu could do more<br>◯ No, my institution provides little to no support<br>◯ Unsure |
| Compared to one year ago, how has your likelihood of continuing to conduct research changed? | ◯ Much more likely to stay<br>◯ Somewhat more likely to stay<br>◯ No change<br>◯ Somewhat less likely to stay<br>◯ Much less likely to stay |
| What factors have contributed to this change? | __________________________________________ |

© 2026 Shalev D et al. JAMA Network Open.

| | |
|---|---|
| Since the beginning of this year, have any of the following impacted your perceived likelihood of continuing to conduct research: (Select all that apply) | ☐ Funding instability<br>☐ Governmental restrictions on research topics<br>☐ Visa/immigration concerns<br>☐ Institutional support changes<br>☐ DEI (Diversity, Equity, and Inclusion) policies and backlash<br>☐ Political interference in science<br>☐ Work-life balance and burnout<br>☐ Other (please specify) |
| Other (please specify): | __________________________________ |

| Section 3: Funding Challenges | |
|---|---|
| Have you personally experienced disruptions in funding due to political or policy changes? | ◯ Yes, significant disruptions<br>◯ Yes, minor disruptions<br>◯ No, but I have concerns<br>◯ No, and I do not have concerns |
| Have any of the following policy changes impacted your research funding or ability to secure grants? (Select all that apply) | ☐ NIH funding limitations or budget cuts<br>☐ Restrictions on specific research areas (e.g., reproductive health, climate science, DEI-related topics)<br>☐ Changes in indirect cost recovery policies<br>☐ Limitations on international collaborations<br>☐ Government-university relations not specific to my own project<br>☐ Other (please specify) |
| Other (please specify): | __________________________________ |

| Section 4: Policy Changes and Research Environment | |
|---|---|
| Have recent or pending legislation or policy changes affected your ability to conduct your research? | ◯ Yes, significantly<br>◯ Yes, minorly<br>◯ No, but I am concerned about future impact<br>◯ No, and I am not concerned |
| If yes, in what ways has policy affected your research? | __________________________________________ |
| How concerned are you about policy changes that may impact academic freedom? | ◯ Extremely concerned<br>◯ Very concerned<br>◯ Moderately concerned<br>◯ Slightly concerned<br>◯ Not at all concerned |
| How concerned are you about policy changes that may impact research funding? | ◯ Extremely concerned<br>◯ Very concerned<br>◯ Moderately concerned<br>◯ Slightly concerned<br>◯ Not at all concerned |

© 2026 Shalev D et al. JAMA Network Open.

| Section 5: Career Plans and Future Directions | |
|---|---|
| Do you plan to apply for an R01 or equivalent within the next three years? | ◯ I have already submitted an R01 or equivalent application.<br>◯ I plan to apply within the next year.<br>◯ I plan to apply within the next 2-3 years.<br>◯ I am unsure whether I will apply.<br>◯ I do not plan to apply. |
| Compared to one year ago, how has your likelihood of applying for an R01 changed? | ◯ Much more likely to apply<br>◯ Somewhat more likely to apply<br>◯ No change<br>◯ Somewhat less likely to apply<br>◯ Much less likely to apply |
| If you are unsure or do not plan to apply for an R01, what are your primary reasons? (Select all that apply) | ☐ Difficulty securing preliminary data<br>☐ Concerns about funding stability<br>☐ Desire to transition out of academic research<br>☐ Institutional barriers<br>☐ Governmental policies unfavorable to science<br>☐ Other (please specify) |
| Other (please specify): | ________________________________ |
| If you were to leave your federally-funded research role, what career paths would you consider? | ☐ Industry<br>☐ Policy/advocacy<br>☐ Non-NIH-funded nonprofit research<br>☐ Science communication<br>☐ Clinical practice<br>☐ Teaching<br>☐ Other (please specify) |
| Other (please specify): | ________________________________ |
| Are there other policy changes that you see as particularly relevant to the future of scientific careers? | ________________________________________ |

| Section 6: Open-Ended Feedback | |
|---|---|
| What support or policy changes would help you feel more secure in continuing your research career? | ________________________________________ |
| Please share any additional comments on how the political climate affects your research and career decisions. | ________________________________________ |
| Which of the following best describes your political views? | ◯ Very liberal<br>◯ Somewhat liberal<br>◯ Moderate<br>◯ Somewhat conservative<br>◯ Very conservative<br>◯ Libertarian<br>◯ Other (please specify)<br>◯ Prefer not to say |

© 2026 Shalev D et al. JAMA Network Open.

**eTable.** Tabulation of factors influencing perceived likelihood of continuing to conduct science

| | |
|---|---|
| Funding instability | 1766 |
| Political interference in science | 1355 |
| Governmental restrictions on research topics | 1166 |
| Work-life balance and burnout | 933 |
| Diversity, Equity, and Inclusion policies and backlash | 891 |
| Visa/immigration concerns | 330 |
| Other | 76 |

Participants could select all that apply. Raw numbers are provided corresponding to the number of respondents who selected each reason

© 2026 Shalev D et al. JAMA Network Open.
