# Supplementary material for: Federal Policy Changes and Career Stability Among NIH K-Award Recipients
Source: JAMA Netw Open. 2026 Apr 22;9(4):e268554. doi: 10.1001/jamanetworkopen.2026.8554 (PMC13103800; doi:10.1001/jamanetworkopen.2026.8554)
Supplement: Supplement 2. — Data Sharing Statement [file jamanetwopen-e268554-s002.pdf]

Data Sharing Statement

Shalev. Federal Policy Changes and Career Stability Among NIH K-Award Recipients. *JAMA Netw Open*. Published April 22, 2026. doi:10.1001/jamanetworkopen.2026.8554

Data
Data available: No

Additional Information
Explanation for why data not available: Because there are certain combinations of award type and geography that can essentially identify an individual, we will only share results in aggregate. However, we would be happy to work with other investigators to support additional analyses conducted by our team with results shared.
